# Retraction: Effect of co-encapsulation using white and red onion peel extract on the viability and stability of *Lacticaseibacillus rhamnosus* under stressful conditions

**DOI:** 10.1371/journal.pone.0327178

**Published:** 2025-06-27

**Authors:** The PLOS One Editors

The *PLOS One* Editors retract this article [1] due to concerns about potential manipulation of the publication process. These concerns call into question the validity and provenance of the reported results. We regret that the issues were not identified prior to the article’s publication.

AE, MA, FA, and MRK did not agree with the retraction. YAA, FS, AAhmed, SW, MUF, and AAsghar either did not respond directly or could not be reached.

## References

[1] Ejaz A, Ali YA, Afzaal M, Saeed F, Ahmed A, Waliat S, et al. Effect of co-encapsulation using white and red onion peel extract on the viability and stability of *Lacticaseibacillus rhamnosus* under stressful conditions. PLoS One. 2025;20(3):e0311952. doi: 10.1371/journal.pone.0311952 40063882 PMC11893123

